# Physicochemical Characteristics of Microplastics Present in Sediments of the Veracruz Reef System National Park Using Nile Red and SEM/EDS Methods

**DOI:** 10.3390/toxics14070610

**Published:** 2026-07-12

**Authors:** Claudia Araceli Dávila-Camacho, Silvia Alejandra Santos-Escobar, María del Refugio Castañeda-Chávez, Magnolia Gricel Salcedo-Garduño, Fabiola Lango-Reynoso

**Affiliations:** Tecnológico Nacional de MéxicoInstituto Tecnológico de Boca del Río, Km 12 Carretera Veracruz-Córdoba, Boca del Río 94290, Veracruz, México; claudia.dc@bdelrio.tecnm.mx (C.A.D.-C.); m23990514@bdelrio.tecnm.mx (S.A.S.-E.); mariacastaneda@bdelrio.tecnm.mx (M.d.R.C.-C.); magnoliasalcedo@bdelrio.tecnm.mx (M.G.S.-G.)

**Keywords:** microplastics, reef sediments PNSAV, Red Nile, SEM/EDS

## Abstract

Microplastics (MP) are emerging pollutants found in the Veracruz Reef System National Park (PNSAV). They are widely distributed in the aquatic environment and are ingested by organisms either accidentally or because they are mistaken for food. In this article, the author analyses the physicochemical characteristics of reef sediments using visual identification, Nile Red (NR), scanning electron microscopy (SEM), and Energy Dispersive Spectroscopy (EDS) of microplastics found in sediments adjacent to reefs; this allowed us to determine their distribution within the PNSAV. A total of 687 microplastics per kilogram of dry sediment were quantified, including fibres (98.5%), films (1.4%), and fragments (0.01%). The predominant colour was transparent (60.2%), followed by blue (16%) and red (12%). Based on their MP distribution, the sites were classified into two groups: north and south. The sampling stations with the highest concentrations of MP are located in the group of reefs south of the PNSAV, with the Anegadilla Reef being the most affected station. SEM/EDS analyses revealed elemental spectra dominated by carbon and oxygen, which is consistent with the expected elemental composition of polymeric materials. Peaks of chlorine were detected in some MP particles, suggesting the possible presence of chlorinated polymers, such as polyvinyl chloride (PVC). In addition, elements such as titanium, silicon, and nickel were identified, which could be associated with inorganic particles adhering to the surface or with additives commonly incorporated during the manufacture of plastics such as polyethylene (PE), polypropylene (PP), polyvinyl chloride (PVC), polystyrene (PS), and polyethylene terephthalate (PET). The presence of elements such as titanium and nickel were detected; these are found in additives used in the manufacturing of polymers.

## 1. Introduction

Microplastics (MP) are among the emerging contaminants in the aquatic environment; due to their density-related properties, they may remain suspended in the water column or settle in sediments. They are transported by marine currents and can be mistaken for food by biota [1].

Because MP are widely distributed in the environment, it is essential to deepen the knowledge on their physicochemical characteristics, their distribution in the sea, and their effects on biological communities through the trophic web, considering their bioaccumulation and their role as vectors of low-molecular-weight chemical substances and other adsorbed contaminants [2,3,4]. Several studies have shown that organisms ranging from plankton to a variety of invertebrates and vertebrates ingest and accumulate MP [5]. MP are potentially toxic to fish, since they adsorb plastic-derived chemical additives, may induce false satiety, and, in severe cases, cause death [6,7].

Likewise, the incorporation and accumulation of plastics and MP within marine biota has been documented along the trophic web, from zooplankton to bivalve molluscs, polychaetes, benthic and pelagic fish, and seabirds [8]. MP have been reported as a threat to marine ecosystems; however, the magnitude of their ecological and ecotoxicological effects continues to be under investigation, given the complexity of the interactions between plastic particles and their associated contaminants [9].

Reef systems such as the PNSAV are ecosystems characterised by high diversity and complexity, providing goods and services to the organisms inhabiting these communities. Nevertheless, the ecological balance of this ecosystem is threatened by anthropogenic activities that promote marine pollution generated by plastics [10]. Coastal research indicates, at a global scale, that marine litter is composed of up to 90% plastic waste [11]. Throughout their pathway toward marine ecosystems, plastics are exposed to several environmental factors such as photodegradation, thermo-oxidative degradation, hydrolysis, thermal degradation, and biodegradation, causing wear and fragmentation of the polymers until they reach millimetre sizes classified as microplastics [9].

MP such as PE, PP, PVC, PS, and PET may enter marine systems [12]. Nevertheless, Polymers are not the only toxic materials entering ecosystems; additives used to improve plastic quality, such as plasticizers, flame retardants, colourants, and lubricants, are also transported [13]. Furthermore, MP have been reported to trigger morphological changes in organisms, growth reduction, disturbances in photosynthetic activity, and an increase in stress-related proteins, promoting immobilisation and mortality in some species [14].

However, Some researchers suggests that plastic debris may also act as artificial substrates for microorganisms and invertebrates, favouring the formation of new microhabitats and local colonisation processes; that is, they may be generating new ecological interactions [15]. For this reason, the physicochemical characteristics of MP found in sediments adjacent to reefs of the PNSAV were analysed.

The physicochemical characterisation of MP is an essential tool to understand their origin, distribution, transport, and potential interaction with biota; therefore, reliable methodologies for their detection and identification must be applied. The PNSAV is a highly biodiverse ecosystem subjected to intense anthropogenic influence derived from port, urban, industrial, fishing, and tourism activities, as well as from continental water inputs. Based on the available evidence, the following hypothesis was proposed: the concentration, distribution, and physicochemical characteristics of the presumed MP present in the reef sediments of the PNSAV vary spatially as a result of the anthropogenic pressures.

The National Oceanic and Atmospheric Administration (NOAA) [16] and the International Organization for Standardization (ISO24187:2023) [17] establish guidelines for the sampling, detection, identification, and quantification of microplastics in soil and water. Within these recommendations, the chemical identification of polymers is usually performed through reference analytical techniques such as Fourier-Transform Infrared Spectroscopy (FTIR), Raman Spectroscopy, and Pyrolysis Gas Chromatography coupled to Mass Spectrometry (Py-GC/MS), owing to their high capacity to identify the polymeric composition of the particles [18,19]. However, point out that these techniques require specialised infrastructure, highly qualified personnel, relatively long analysis times, and high operating costs, which may limit their routine application in studies involving large numbers of environmental samples [12]. In this context, fluorescence staining with Nile Red (NR) has emerged as a rapid, sensitive, and cost-effective screening tool for the preliminary detection of presumed microplastic particles, allowing the selection of particles of interest for subsequent characterisation by complementary analytical techniques such as FTIR or Raman [13,14,20,21,22].

The NR staining method 9-(diethylamino)-5H-benzo[a]phenoxazin-5-one shows a high affinity for hydrophobic materials, including several plastic polymers, enabling rapid detection through fluorescence, with a potential effectiveness of up to 80% for MP determination [22]. This staining technique does not interfere with subsequent analytical methods used to confirm the type of polymer in the samples, and is therefore recommended by [18,19] for MP characterisation and quantification. For this reason, the technique has been used as a screening approach, since it detects presumed microplastic particles that require subsequent confirmation through spectroscopic techniques.

Scanning Electron Microscopy (SEM) characterises morphology; due to its high resolution, it enables the analysis of numerous particles more rapidly and efficiently, reducing the possibility of confusion with materials exhibiting features similar to polymers. Observation at higher resolution reveals textures such as grooves, indentations, and weathering along the surface [19,23].

In contrast, Energy Dispersive Spectroscopy (EDS) determines the elemental composition of the material present in the sample under study. EDS does not identify polymers directly; it serves as a complementary technique, detecting carbon and oxygen, chlorine peaks characteristic of chlorinated products, and chemical elements associated with additives used in polymer production [23].

In this context, the objective of this research was to characterise the presence of potential MP in reef sediments of the PNSAV, integrating the abundance, spatial distribution, and main physicochemical characteristics morphological evidence (SEM), elemental evidence (EDS), and fluorescence (NR), with the aim of generating information that contributes to understanding their dynamics and their potential role as a vector of contamination and chemical substances present in the ecosystem, providing a baseline for future ecological risk assessments, monitoring, and conservation strategies for the PNSAV.

## 2. Materials and Methods

### 2.1. Study Area

**Figure 1:**
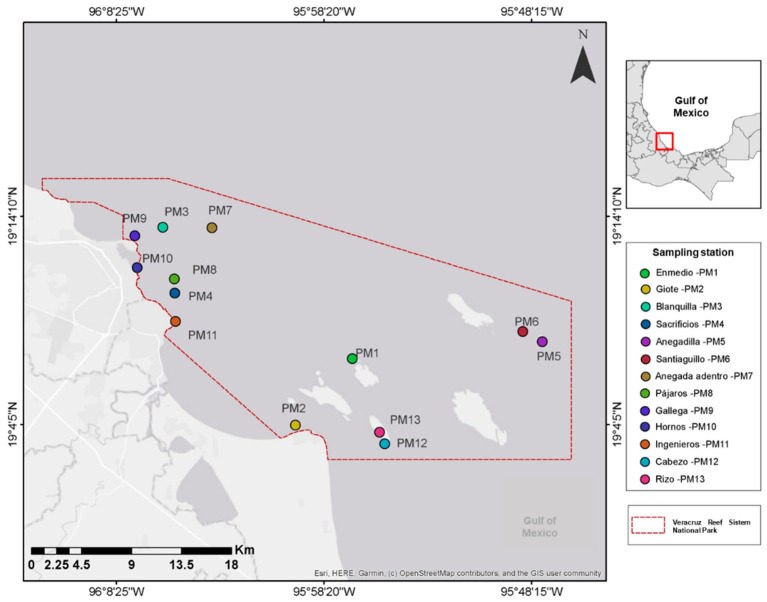
Location of the PNSAV, the research area, and sampling sites.

The PNSAV is located in the central-southern portion of the Gulf of Mexico. It is a Natural Protected Area situated off the coast of the municipalities of Veracruz, Boca del Río, and Alvarado, between the geographic coordinates 19°02′24″ and 19°16′00″ north latitude and 95°46′19″ and 96°12′01″ west longitude, covering an area of 65,516.47 hectares. It includes fringing reefs close to the shoreline, reef lagoons, shoals, cays, and beaches, comprising 45 reefs (Figure 1) [24]. The reefs of the PNSAV exhibit high resilience to the input of freshwater and sediments from the La Antigua, Jamapa, and Papaloapan rivers, located to the north, centre, and south of the park, respectively. The reefs can be divided into two main groups: the first, to the north, comprising smaller reefs closer to the shore, and the second, to the south, comprising larger reefs located further offshore. For this study, reefs were selected according to their geographical location.

### 2.2. Sampling

Sediment sampling was carried out by SCUBA diving. Descents were performed in pairs, with a main diver and a support diver; total immersion time varied depending on the depth and characteristics of the site. At the deeper sites, sediment was collected using a metallic sampler with a diameter of 19 cm and a height of 7 cm, following [25].

To perform sediment sampling on the reef substrate, the recommendations of [26] were considered; surface sediment samples were collected by a diver using a corer. The following precautions were taken:

- Approaching the site from the downstream direction of the current to avoid contaminating the sample with suspended material.
- In the event of a slow current, it was decided to wait in order to reduce the suspended sediment.
- Avoiding the resuspension of sediments.
- Preserving the fine-grained material.

### 2.3. Quality Assurance and Quality Control (QA/QC)

In accordance with international guidelines [26,27,28], and to try to minimise the risk of cross-contamination by presumed MP during sample processing, the recommended Quality Assurance (QA)/Quality Control (QC) measures were followed. In the laboratory, glassware was used, washed, rinsed with distilled water, and covered with aluminium foil until use. During sample processing, cotton clothing, nitrile gloves, and metallic tweezers were used. The solutions employed were prepared and stored in glass containers. Samples were kept covered with aluminium foil at all times to avoid the probability of contamination by airborne particles; likewise, air currents and the use of air conditioning in the laboratory were avoided.

As a contamination control measure, blanks were processed following the same protocol used for the samples, including the reagents employed during digestion and Nile Red staining. The blanks were examined under the same microscopic conditions as the samples, and no fluorescent particles resembling MP were identified; therefore, no corrections were required in the counts obtained.

### 2.4. Sample Processing

At the Aquatic Resources Research Laboratory (LIRA) of the Instituto Tecnológico de Boca del Río, Veracruz, wet sediment samples from thirteen reefs of the PNSAV were processed, using one kilogram of sample per sampling site. For the moisture-reduction process, a Felisa fe-291ad drying oven was used at a constant temperature of 60 °C [29]. A total of 7.5 kg of dry sediment was analysed. Organic matter was degraded by the addition of 30% hydrogen peroxide (H_2_O_2_) and 6% sodium hexametaphosphate ((NaPO_3_)_6_). Distilled water was then added to stop the reaction, and the samples were subsequently left to stand for 24 h [16,29]. To complete sample preparation, a drying process was carried out at 60 °C for 24 h. The dry sediment was separated using a series of sieves with different mesh openings, selecting the sizes within the range of MP (0.063 mm, 0.5 mm, 0.9 mm, 1.1 mm, 2.3 mm, and 5 mm) [30].

Density separation was carried out using a saturated sodium chloride (NaCl) solution (ρ ≈ 1.2 g/cm^3^), following the NOAA laboratory protocol [16,30,31,32]. The mixture was subjected to constant manual agitation for five minutes; it was then allowed to settle for one hour to extract the largest possible amount of MP [30,32]. Floating particles were filtered with the assistance of an Arsa AR-1500L vacuum pump, using microcellulose membranes, which were rinsed with distilled water and subsequently dried at 50 °C for ten minutes. The dry samples were stored in glass Petri dishes to avoid contamination. The samples obtained were observed under a stereoscopic microscope at 40×; MP were identified and classified according to their shape, colour, and size [33].

### 2.5. Nile Red (NR) Staining Technique

To stain the MP, 1 mL of NR solution was used (10 μg of the dye dissolved in 1 mL of acetone); subsequently, using a Pasteur glass pipette, drops of the solution were applied directly onto the particles previously selected under optical microscopy until the filter was completely moistened. The filters were placed in a glass Petri dish to avoid cross-contamination; the Petri dish was covered with aluminium foil to keep it in the dark and prevent photobleaching [34,35]. In addition, the methodology proposed by [36] was applied; that is, the samples were dried at a constant temperature of 60 °C for 10 min. For fluorescence-based visualisation and detection, a high-intensity ultraviolet (UV) lamp was used, with a UV-A long-wave spectrum (365–405 nm), adjustable gooseneck, and which is portable and rechargeable. Optical analysis of the stained polymers was performed with a Stereo VE-s3 stereoscopic microscope at 40x magnification.

### 2.6. Scanning Electron Microscopy and Energy Dispersive Spectroscopy (SEM/EDS) Analysis

SEM/EDS analysis was performed using a JEOL JSM-7600-F field-emission scanning electron microscope. The MP were placed on a double-sided carbon tape and adhered to a copper base to be introduced into the vacuum chamber of the equipment. The samples were kept under vacuum for eight hours before analysis; subsequently, SEM analysis and photographic imaging of the surface of the materials were carried out. EDS analysis was performed during electron irradiation of the sample, allowing the acquisition of the X-ray signals of the elements present in the sample [37].

### 2.7. Statistical Analysis

Descriptive statistics of the data were computed, as well as the correlation analysis between MP concentration and size, using Excel^®^ software; one-way Analysis of Variance (ANOVA) was performed with Past version 3^®^ software. The database was organised by sampling station (n = 13), recording the abundance of presumed MP expressed as number of particles per kilogram of dry sediment; descriptive statistics (mean, standard deviation, and frequencies) were computed. The response variables were: total concentration of presumed MP (particles kg^−1^ of dry sediment); differences in abundance; dominant shapes; abundance by size classes; and abundance by colour; these were evaluated through one-way ANOVA. When ANOVA revealed significant differences (*p* < 0.05), Turkey’s multiple comparisons test was applied to identify the groups that differed from one another. Likewise, correlation analyses were carried out between the number of particles per kilogram of sediment and the environmental variables considered in the study. A significance level of α = 0.05 was used in all cases. Normality assumptions were assessed by the Shapiro–Wilk test, as suggested by [32].

## 3. Results

### 3.1. Classification of MP by Their Physical Properties

The behaviour of the data was analysed, and it was determined that statistically significant differences existed among the mean concentrations of the observed data.

A total of 5155 presumed MP pieces were observed across the thirteen sampling stations, with an average of 396 pieces per station, representing an estimated average of 687 presumed plastic particles (MP) per kilogram sampled (7.5 kg of dry sediment analysed) (Table 1).

It is worth noting that the reef sediments of the PNSAV displaying the highest MP concentrations were the reefs located farther from the coast, at the southern and northern ends. Sacrificios was the sampling site with the lowest MP concentration. The highest observed abundance corresponded to Anegadilla, which exhibited the highest MP concentration (756 pieces) and was the reef with the highest concentration of microfibres. In contrast, at Sacrificios, 145 microfibres were counted, making it the station with the lowest concentration (Table 1).

#### 3.1.1. Characterisation of MP by Colour

The following colours were observed: transparent (60.2%), blue (16%), red (12%), black, brown, green, yellow, and purple. The ANOVA yielded F = 10.73, df = 36.96, and *p* < 0.001; therefore, a Tukey test was performed, revealing that the transparent colour showed statistically significant differences (*p* < 0.05).

The abundance of transparent particles was significantly higher than that of the remaining colours, whereas no statistical differences were detected among the other colours. A total of 3104 transparent MP pieces were counted; the reef with the highest concentration of transparent MP was Anegadilla, with 572 pieces, representing 73% of the total concentration; conversely, Santiaguillo yielded 81 transparent MP, being the station with the lowest concentration (Table 2).

#### 3.1.2. Characterisation of MP by Shape

Three MP shapes were identified: fibres (98.5%), films (1.4%), and fragments (0.01%); by comparing the *p*-values obtained, it was observed that fibres are the most abundant particles in the results, since they exhibited significant differences (*p* < 0.001) with respect to fragments and films.

A Tukey test was performed with a 95% confidence level; the *p*-values showed significant differences for fibres vs. fragments (*p* = 7.00 × 10^−9^) and non-significant differences for fragments vs. films (*p* = 0.9927), whereas fibres vs. films (*p* = 9.81 × 10^−9^) did show a significant difference; that is, the abundance of fibres was significantly higher than that of the other two MP types (Table 3).

#### 3.1.3. Characterisation of MP by Size

MP were examined on a scale of 1 mm to 5 mm using ANOVA: F (4,60) = 5, *p* = 0.0015. The size distribution was not homogeneous; therefore, significant differences exist between size 1 and sizes 3, 4, and 5. A Tukey test was performed to identify the groups exhibiting differences. MP within the 1–2 mm range showed no significant differences (*p* > 0.05) but did show considerable differences relative to the 3–4 mm ranges (Table 4).

The El Giote sampling station displayed the highest concentration within the most representative ranges: 1 and 2 mm, with 370 and 240 pieces, respectively; in contrast, at Anegadilla, only eight pieces of 1 mm and 22 pieces of 2 mm were counted, being the station with the lowest concentration for both size classes among the reefs of the southern group. Therefore, it is concluded that the groups exhibiting the highest concentrations in the analysed samples are the smaller MP, within the 1 and 2 mm range.

### 3.2. Determination of the Physicochemical Composition of MP

To perform the physicochemical characterisation of the microplastic particles present in the study area, two techniques were selected: optical identification based on the fluorescence of MP with dyes compatible with hydrophobic materials such as NR, and the analysis of MP components by SEM/EDS.

#### 3.2.1. MP Staining Using NR

**Figure 2:**
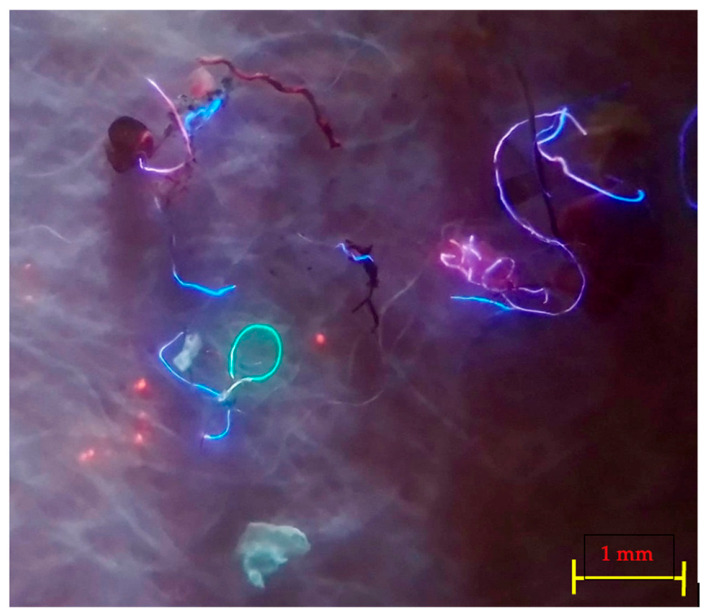
Observation of different lengths of fluorescence emission wavelengths of tiny particles displaying fluorescence and microfibres stained with NR under UV light excitation.

**Figure 3:**
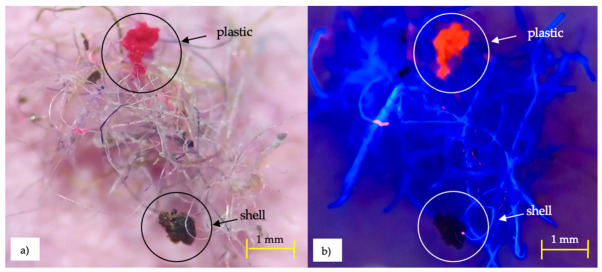
Observation of fluorescence of different colours: (**a**) conventional optical microscopy image showing a fragment of a possible polymer and a piece of shell, and (**b**) microfibres stained with NR under UV light excitation, visible with blue fluorescence.

Under this technique, five reef sampling stations were analysed: La Gallega, El Giote, Sacrificios, Enmedio, and Santiaguillo. After staining the MP with NR and exposing them to UV light, three fluorescence colours were visualised and confirmed through conventional optical microscopy: blue fluorescence was observed at an excitation wavelength of 365 nm; green-yellow ranges displayed an emission wavelength of 445 nm; red-orange fluorescence was visible at excitation wavelengths between 450 and 490 nm; and blue fluorescence with emission between 516 and 565 nm was the most frequent. During microscopic observation, fluorescent particles with very small dimensions compatible with polymeric material were detected; however, due to the resolution limit of the optical microscope employed, it was not possible to confirm whether these particles corresponded to nanoplastics. Consequently, the presence of nanoplastics cannot be established with certainty through the methodology applied (Figure 2 and Figure 3).

#### 3.2.2. Scanning Electron Microscopy and Energy Dispersive Spectroscopy (SEM/EDS)

**Figure 4:**
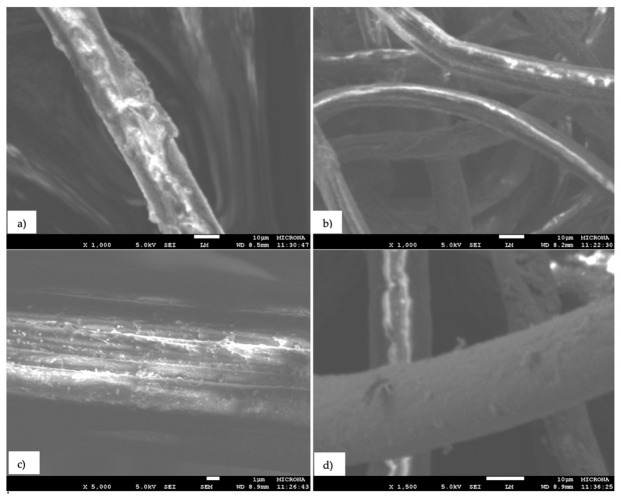
Surface morphologies observed in the presumed microplastic fibres under SEM at 1000x: (**a**) fragmentation of the material; (**b**) smooth surface; (**c**) microfibre with grooves observed at 5000x; (**d**) microfibre with adhered particles observed at 1500x.

Plastic particles from three sampling stations Anegadilla, Anegadilla de Adentro, and Hornos, were analysed using the SEM/EDS analytical technique with a JEOL JSM-7600-F instrument. The SEM analysis revealed surface textures present on the microfibres (Figure 4); that is, surface fragmentation of the material was observed (Figure 4a); in Figure 4b, the surface of the fibre appears smooth, without wear or fragmentation of the material. The most frequently observed surface modifications were grooves (Figure 4c); they exhibited long, shallow indentations around the fibre. Another recurrent change was the adherence of particles onto the plastic surface (Figure 4d), generating a mineral layer on the microfibres.

**Figure 5:**
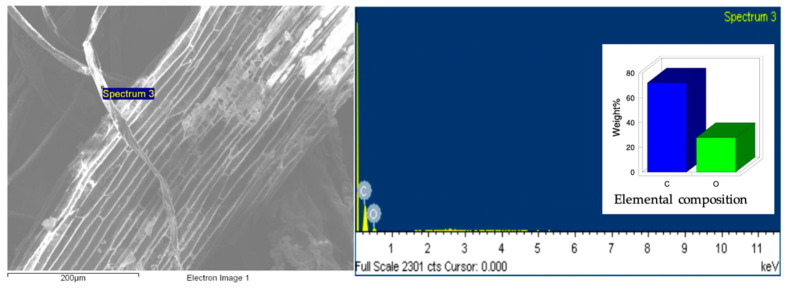
Elemental composition of MP, characteristic of polymers with carbon peaks, obtained though EDS.

**Figure 6:**
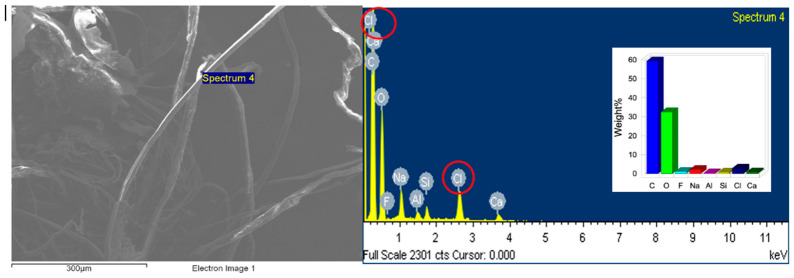
The chlorine peaks marked with red circles characteristic of chlorinated plastics such as polyvinyl chloride (PVC).

In the EDS analysis, punctual carbon peaks were observed (Figure 5), which are characteristic of materials composed of polymers, providing evidence of MP in the analysed samples. In some samples, significant chlorine peaks were obtained, which are representative of chlorinated MP such as PVC; the presence of representative chlorine peaks suggests the occurrence of PVC-based MP (Figure 6).

**Figure 7:**
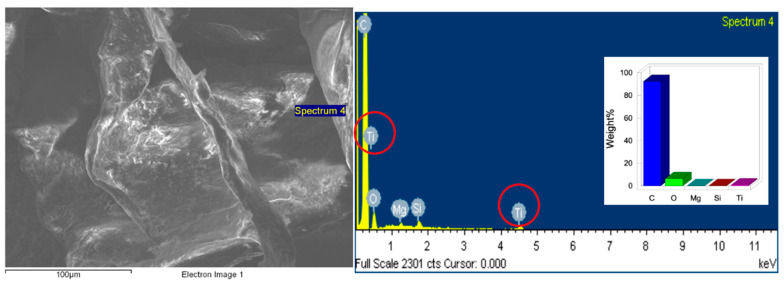
Titanium spectral peaks, marked in red, are observed in EDS analysis of polymers.

The sampling station that displayed the appearance of chlorine spectral peaks is Anegadilla, which is the station with the highest MP concentration among all the analysed samples. Samples exhibiting a distinctive brightness were observed; when examined through EDS, adhered mineral particles of titanium were identified (Figure 7).

Through EDS, false positives were ruled out; when less representative carbon peaks and higher concentrations of calcium and silicon were observed, the physical structure and elemental composition dispels the belief that it is made of plastic. The presence of abundant calcium peaks in samples containing lower carbon concentrations confirms that the material corresponds to organic matter, with characteristics of shells, which are abundant at the analysed sampling stations.

### 3.3. Statistical Analysis

From the descriptive statistics, the following was obtained: Anegadilla was the reef with the largest number of particles by colour, with 780 pieces. The transparent colour was the most abundant, with 3104 pieces; Anegadilla was the reef with the highest number of transparent pieces (572). The purple colour was the least represented, with 12 pieces. The lowest standard deviation (SD) = 27 in the distribution of particles by colour was found at Santiaguillo. The highest SD = 197.3 in the distribution of particles by colour was found at Anegadilla.

The fibre type was the most abundant across the entire PNSAV, with 5077 pieces. Sacrificios had the lowest number of particles by type, with 146 pieces. The highest SD = 433 in the distribution of particles by type was found at Giote. Regarding filaments, 76 pieces were detected, and two pieces were of the fragment type. The lowest SD= 83.4 in the distribution of particles by type was found at Sacrificios.

Size 1 was the most frequent, with 2255 pieces, and Giote was the reef with the highest number of pieces (370). Size 3 was the least frequent, with 507 pieces.

The lowest SD = 20.6 in the distribution of particles by size was found at Sacrificios. The highest SD = 184.7 in the distribution of particles by size was found at Anegadilla.

To verify whether the dataset followed a normal distribution, the Shapiro–Wilk test was applied, and Levene’s test was performed to assess the homogeneity of variances among groups at each sampling point to select the most appropriate statistical procedure.

- For colour: Shapiro W = 0.665, *p* = 2.056 × 10^−6^; no homogeneity was found.
- For shape: Shapiro W = 0.752, *p* = 1.495 × 10^−6^; no homogeneity was found.
- For size: Shapiro W = 1.70 × 10^−8^, *p* = 0.071; homogeneity was found.

That is, for the colour and shape variables, variances were significantly different among the evaluated categories (*p* < 0.05), and the assumptions of normality and homogeneity were not met. In contrast, for the size variable, no significant differences in variances were detected (*p* = 0.071), indicating that this assumption was fulfilled only for the latter variable.

Subsequently, the abundance of presumed MP was used as the observation unit for each sampling station and for each category: colour, shape, and size. For the colour and shape variables, since the data did not meet the assumptions of normality and homogeneity of variances, in addition to the one-way ANOVA, the non-parametric Kruskal–Wallis test (*p* > 0.05) was applied for colour and shape. That is, the colours do not exhibit the same abundance. The F-value for shape indicates that between fibres, fragments, and films, there is substantial variability among stations. For size, differences exist, but to a lesser degree than in the two previous groups (Table 5).

A Tukey multiple comparisons test was performed (*p* < 0.001); through this test, the categories responsible for the differences detected by the ANOVA were identified. Significant differences were detected in the colour category for the transparent group. For shapes, the result indicates that fibres show the highest dominance, and for size, differences are moderate but smaller.

For η^2^ = 0.694, 69.4% of the total observed variability is explained by shape.

Correlations between quantitative variables were assessed using Pearson’s coefficient when both variables met normality, and Spearman’s coefficient otherwise. A significance level of α = 0.05 was used. The significance level was null for both tests.

## 4. Discussion

The distribution of MP could be influenced by anthropogenic activities such as tourism, fishing, maritime transport, and continental water discharges from the La Antigua, Jamapa, and Papaloapan rivers, as well as by marine currents. Studies on the hydrodynamics of the PNSAV have described the transport of particles due to current rectification induced by bathymetric changes. In the central part of the PNSAV, cyclonic and anticyclonic eddies develop, preventing particles suspended in the water column from settling; this is consistent with the observation that Sacrificios was the sampling point with the lowest MP concentration [32,38]. Therefore, the highest abundance observed at Anegadilla could be related to hydrodynamic processes and continental inputs previously described for the PNSAV.

The MP abundance estimated in this study differs from the results obtained by [39] in reef sediments from Lombok, Indonesia, where tourism and maritime traffic activities also occur; they reported 77 plastic particles per kilogram of sediment. Their results represent only 11% of the concentration obtained in the present study for the Papaloapan area. The observed distribution could be caused by the influence of the three rivers converging in the PNSAV; this condition can lead to higher MP concentrations due to the transport of particles by continental waters, as stated by [34,40].

The transparent colour was the most predominant (60.2%); in agreement with several studies among them, those mentioning that blue and transparent colours were the most abundant in their results, which increases the risk of ingestion arising from the preference of organisms for light-coloured polymers. Transparent fibres represent a pattern observed in several studies; these fibres may originate from synthetic textiles, fishing gear, or result from photodegradation and weathering [41]. The present study did not identify the specific origin of the polymers. This MP colour could be accidentally ingested and adsorb larger amounts of toxic contaminants, such as dioxins, pesticides, flame retardants, and polychlorinated biphenyls (PCB), which cause liver damage in fish [42,43].

MP within the 1 to 2 mm range presented the highest concentration, coinciding with the results of [19]; fibres displayed the highest abundance. Several authors report that microfibres are predominant in studies conducted in marine and coastal environments [18,19]. This predominance is attributed to the continuous input of synthetic fibres derived from wastewater, textiles, fishing activities, and other anthropogenic sources [4]. However, these results do not agree with those obtained by [32], who reported that foams were the most frequently observed MP in their results; in the PNSAV, no foams were recorded.

The shape and size favoured the infiltration of plastic particles through the porosity of the reef sediments, promoting a greater accumulation of MP on the seabed [34]. However, physical characteristics are not the only driver of MP accumulation; the behaviour of cyclonic and anticyclonic currents, the Campeche gyre, and the bathymetry of the PNSAV also promote lower MP concentrations in the reefs of the northern group despite their proximity to the coastal zone, in line with the results reported by [40,44].

The fluorescence ranges observed under the NR staining technique ranged between emission wavelengths of 445 nm (blue) and 516–565 nm (yellow–green and red-orange). In this study, microfibres displaying blue fluorescence exhibited the highest concentration; in agreement with [18], MP with blue fluorescence originate from plastic materials such as polyester, nylon, and polyethylene terephthalate (PET). Researchers such as [9] report that polymers such as polyester, nylon, and PET are present in clothing, fishing gear such as nets and traps, as well as in beverage bottles, which is consistent with the anthropogenic activities carried out in the study area. Green and orange fluorescence peaks were also obtained, similar to the results of the researchers in [36], who confirmed through Fourier-Transform Infrared Spectroscopy (FTIR) that MP emitting green and orange fluorescence corresponded to polymers such as LDPE, HDPE, PP, EPS, PC, PU, EVA, PVC, nylon, and polyester.

In the present work, the SEM analysis allowed the observation of physical changes on the surfaces of the MP: cracks, grooves, roughness, and adhered particles. These features have been described by other authors in both experimental ageing studies and field studies, which have indicated that such surface modifications are associated with the weathering of the material, including prolonged exposure to sunlight (UV rays), marine currents, mechanical abrasion, and marine salinity, and who have reported physical changes on the surfaces of some microfibres, produced by prolonged exposure to environmental conditions [19,23,45,46].

In the EDS analysis, discrete carbon and oxygen peaks were observed, coinciding with what was reported by [47], where the spectra of these elements are described as the fingerprints of polymers. The presence of chlorine was recorded; this element is present in chlorinated polymers such as PVC [23]. Titanium dioxide was also identified; it is a synthetic material used for boat coatings. The results obtained were similar to those identified by [29]; likewise, the presence of nickel and molybdenum was observed, which are used as additives due to their resistance to high temperatures and corrosion in polymer production. The presence of lead, nickel, chromium, copper, zinc, and arsenic is related to the additives used in polymer manufacturing. It has been confirmed that MP are potentially toxic, exhibiting both physical and chemical toxicity to fish, transferring adhered contaminants and chemical substances to their tissues by adsorbing addictive substances and toxic monomers as observed in the present EDS analysis, where some MP particles displayed adhered substances such as chlorine, and metals such as silicon and titanium [6].

In some samples, high calcium peaks were obtained; this result agrees with those of [23]. The authors suggest that the calcium originates from the microbiota encrusted on the surface of the material, which adheres to the plastic surface once the roughness generated by weathering has developed.

## 5. Conclusions

In this research, the physicochemical classification of the MP present in reef sediments of the PNSAV was analysed; the physical results suggest that transparent fibres within the 1 to 2 mm range are the MP with the highest concentration in the study area. Due to their physical condition, they favour the presence of plastic particles in sediments, which promotes greater bioavailability and increases the ecological risk for the organisms. It is suggested that the river mouths influencing the coastal zone of the PNSAV may represent a source of MP contamination; however, the natural conditions of the currents and the bathymetry of the park allow MP to concentrate to a larger extent in the reefs located farther from the coast. The SEM/EDS analyses provided complementary physicochemical evidence for the characterisation of particles compatible with polymeric materials.

SEM analysis allowed for the observation of the physical features causing wear such as indentations, fractures, and weathering which enable the adhesion of organic matter and chemical elements to the plastic surface. Through EDS analysis, elemental evidence compatible with polymeric materials was obtained via discrete carbon and oxygen peaks; chlorinated polymers were identified, as well as titanium and silicon spectra, which are used in additives for the manufacture of synthetic pigments such as titanium dioxide. This confirms that MP pose a threat to marine biota, not only due to the polymers with which they are manufactured, but also because of the additives used to enhance the material, and additionally because of the release of other chemicals during their degradation.

MP are potentially vectors of persistent organic pollutants such as PCB. The present study did not evaluate the presence and effects of these compounds, and therefore this may represent an environmental implication to be investigated in future work.

## Figures and Tables

**Table 1:** Abundance of microplastics (pieces) per sampling station, classified by colour, shape, and size. T = Transparent, B = Blue, R = Red, Bl = Black, Br = Brown, G = Green, Y = Yellow, P = Purple, Fib = Fibres, Frag = Fragment.

Reef	Colour								Type			Size (mm)					Abund. (Pieces)
	T	B	R	Bl	Br	G	Y	P	Fib	Frag	Film	1	2	3	4	5	
Enmedio	497	89	17	30	0	0	3	0	631	0	5	325	184	47	30	25	636
Giote	266	214	200	25	0	8	36	1	750	0	0	370	240	81	32	25	750
Blanquilla	163	57	70	77	8	0	14	0	389	0	0	291	63	19	16	0	389
Sacrificios	89	39	9	6	1	1	1	0	145	0	1	48	53	23	5	17	146
Anegadilla	572	141	31	2	6	20	8	0	756	2	22	8	22	52	424	274	780
Santiaguillo	81	30	31	7	11	0	5	1	161	0	5	89	42	19	9	7	166
Anegada de adentro	427	75	47	0	0	0	0	0	549	0	0	349	114	42	25	19	549
Pájaros	161	30	10	2	0	1	0	1	203	0	2	83	63	20	11	28	205
Gallega	137	47	26	10	14	2	6	3	236	0	9	143	59	20	11	9	245
Hornos	254	51	20	0	0	0	0	0	317	0	8	209	64	29	13	10	325
Ingenieros	140	71	57	8	2	3	9	2	281	0	11	164	52	30	30	16	292
Cabezo	198	91	73	18	3	62	8	4	453	0	4	41	47	110	85	174	457
Rizo	119	44	34	5	3	1	9	0	206	0	9	135	57	15	3	5	215
TOTAL	3104	979	625	190	48	98	99	12	5077	2	76	2255	1060	507	694	609	5155

**Table 2:** Summary of *p*-values from Tukey’s test showing significant differences in the colours present in the samples.

Comparison	*p* Adjusted
transparent—blue	6.12 × 10^−8^
transparent—yellow	4.28 × 10^−10^
transparent—rojo	7.57 × 10^−10^
transparent—black	4.29 × 10^−10^
transparent—brown	4.28 × 10^−10^
transparent—green	4.28 × 10^−10^
transparent—purple	4.28 × 10^−10^

This analysis confirmed that the statistical pattern is dominated by the predominance of transparent particles, while the remaining colours displayed comparable abundances among themselves.

**Table 3:** Tukey test *p*-values identifying significant differences among the shapes present in the sample.

	Fibres	Fragments	Films
fibres	-	7.00 × 10^−9^	9.81 × 10^−9^
fragments	11.15	-	0.9927
films	10.99	0.1626	-

**Table 4:** Tukey test *p*-values, identifying significant differences among the sizes of the microplastics present in the samples.

Size	*p*
size 1 vs. size 1	0
size 1 vs. size 2	0.7761
size 1 vs. size 3	0.002617
size 1 vs. size 4	0.009198
size 1 vs. size 5	0.005258

**Table 5:** Kruskal–Wallis results for each data group.

Factor	ANOVA F
colour	21.320
form	40.855
size	5.001

## Data Availability

The original contributions presented in this study are included in the article. Further inquiries can be directed to the corresponding author.

## Abbreviations

PM	Microplastic
PNSAV	Veracruz Reef System National Park
SEM	Scanning Electron Microscopy
EDS	Energy Dispersive Spectroscopy
PE	polyethylene
PP	polypropylene
PVC	polyvinyl chloride
PS	polystyrene
PET	polyethylene terephthalate
NOAA	National Oceanic and Atmospheric Administration
ISO	International Organization for Standardization
LIRA	The samples were processed at the Aquatic
NR	Nile Red
